# Interstitial Granulomatous Dermatitis as the Initial Manifestation of Granulomatosis with Polyangiitis

**DOI:** 10.7759/cureus.42293

**Published:** 2023-07-22

**Authors:** Ahmad Rimawi, Michael Neinast, Abrahim Rimawi

**Affiliations:** 1 Internal Medicine, Southern Illinois University School of Medicine, Springfield, USA; 2 Internal Medicine, University of Sharjah College of Medicine, Sharjah, ARE

**Keywords:** renal failure, rash, autoimmune disorders, glomerulonephritis, vasculitis

## Abstract

Interstitial granulomatous dermatitis (IGD) is a rare dermatological disorder. It is most commonly associated with autoimmune disorders mainly lupus and rheumatoid arthritis. It has rarely been reported to present as a first manifestation of an underlying vasculitis. Here, we present a case of a 44-year-old woman who presented initially with a violaceous rash starting in her neck and trunk and extending to her limbs, but sparing the palms and soles. She was also found to have an acute kidney injury. A biopsy of the skin lesion confirmed the diagnosis of IGD, and a kidney biopsy showed findings consistent with granulomatosis with polyangiitis. To the best of our knowledge, this is the seventh reported case of IGD associated with systemic vasculitis.

## Introduction

Interstitial granulomatous dermatitis (IGD) is a rare dermatological disorder that presents clinically with violaceous papules or plaques with a diverse distribution [1]. It is most associated with autoimmune diseases and, in particular, rheumatoid arthritis and lupus [2]. It has been rarely reported as a first presentation for granulomatosis with polyangiitis. Here, we report a case of a 44-year-old woman presenting with a skin rash. Histology of the biopsied skin tissue revealed IGD, and she was later diagnosed with granulomatosis with polyangiitis. 

## Case presentation

**Figure 1:**
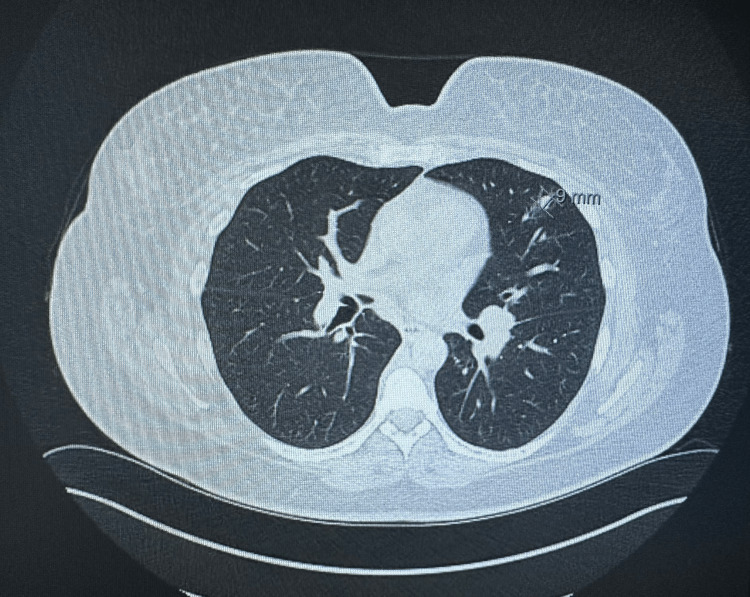
Computed tomography of the chest showing a pulmonary nodule of 9 mm in size.

**Figure 2:**
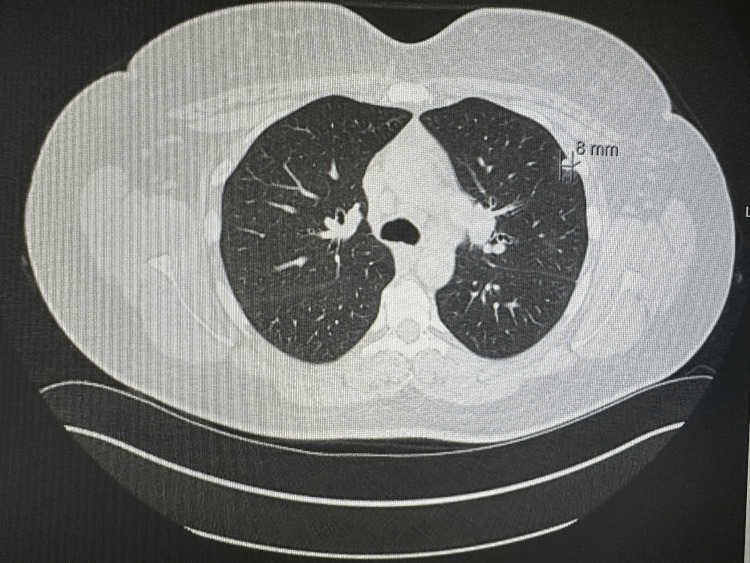
Computed tomography of the chest showing a pulmonary nodule in the left lung of 8 mm in size.

**Figure 3:**
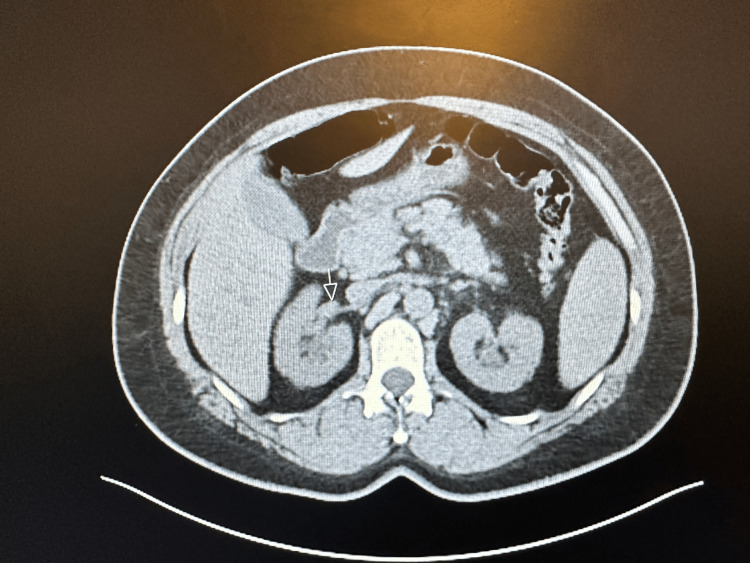
CT-Abdomen showing the right renal pelvis and proximal ureteral thickening.

**Figure 4:**
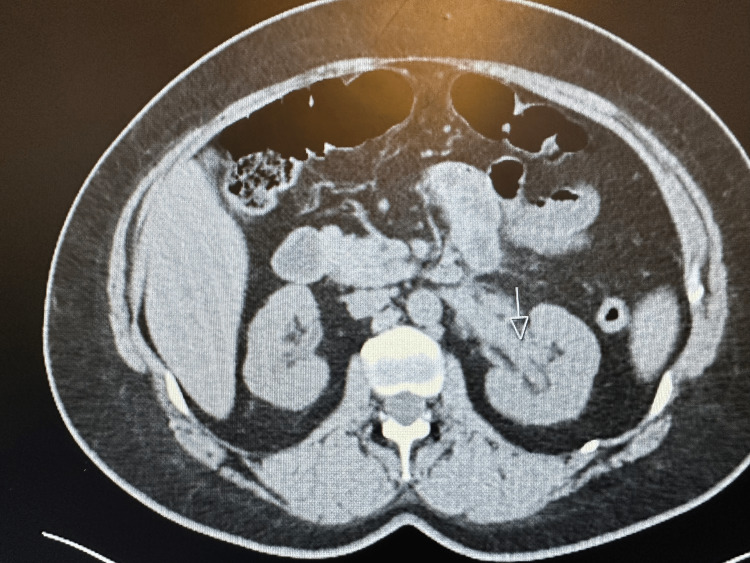
CT-Abdomen showing the left renal pelvis and proximal ureteral thickening.

The patient is a 44-year-old Caucasian woman who presented to the hospital for a new rash eruption of one-week duration. She has a past medical history of hypertension only, and her only medications are lisinopril and metoprolol. One week before presentation, she developed an erythematous urticarial-appearing rash that started on her neck before spreading to her trunk and proximal arms and legs sparing the palms and soles. The rash was non-pruritic and mildly tender to palpation. After two days after her rash eruption, she developed left flank pain that was non-radiating and not associated with dysuria, hematuria, or urinary frequency. She also reported low-grade fevers, malaise, and fatigue. She also reported multiple episodes of epistaxis that started two-three days prior to admission. Upon presentation, she was afebrile and tachycardic with a heart rate of 105 and blood pressure of 135/76. A physical exam showed several pink erythematous annular papules and plaques with central clearing scattered across the anterior and posterior neck, upper back, bilateral arms, and proximal lower extremities. The rest of the physical exam was unremarkable. Upon admission, a complete blood count revealed a hemoglobin of 9.6 gm/dl, white blood cells of 9.3, and platelets of 354. Her basic metabolic profile showed normal electrolyte levels and elevated creatinine at 2 gm/dl (baseline creatinine = 1). She also had elevated inflammatory markers, including an erythrocyte sedimentation rate of 30 and elevated C-reactive protein of 10. Computed tomography (CT) of the chest showed new pulmonary nodules in the left lung, which are new compared to previous imaging (Figures 1-2). Abdominal CT was also done and showed thickening of the bilateral renal pelvis and proximal ureters (Figures 3-4).

**Figure 5:**
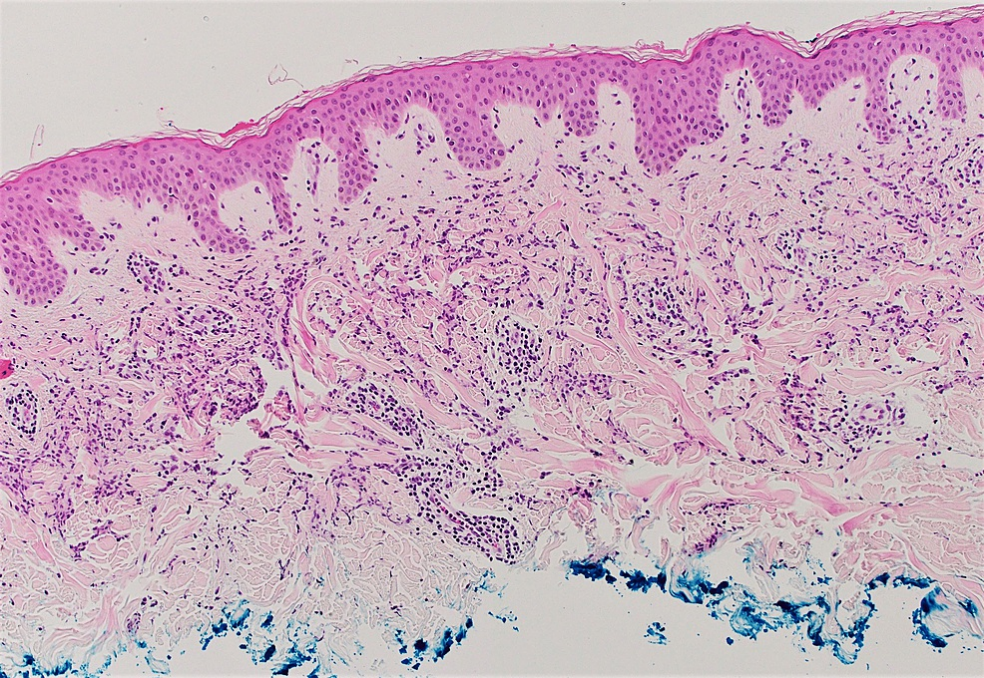
Pathology image of the skin biopsy showing an interstitial dermal histiocytic infiltrate.

**Figure 6:**
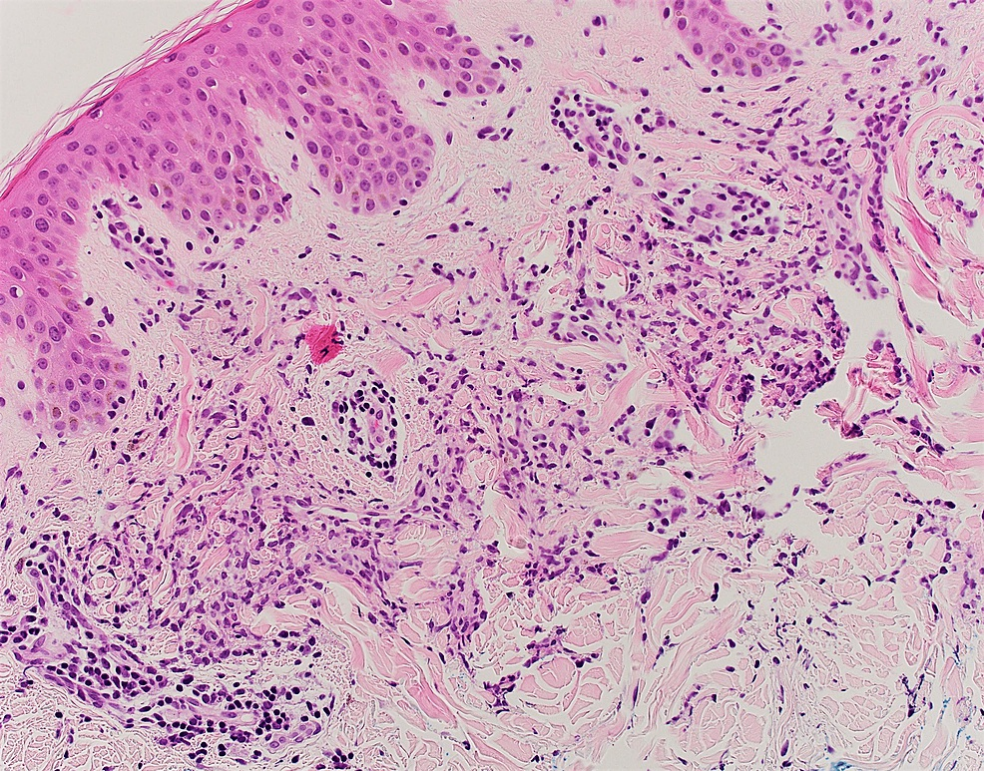
Pathology section showing an interstitial dermal histiocytic infiltrate, consistent with interstitial granulomatous dermatitis with higher magnification.

**Figure 7:**
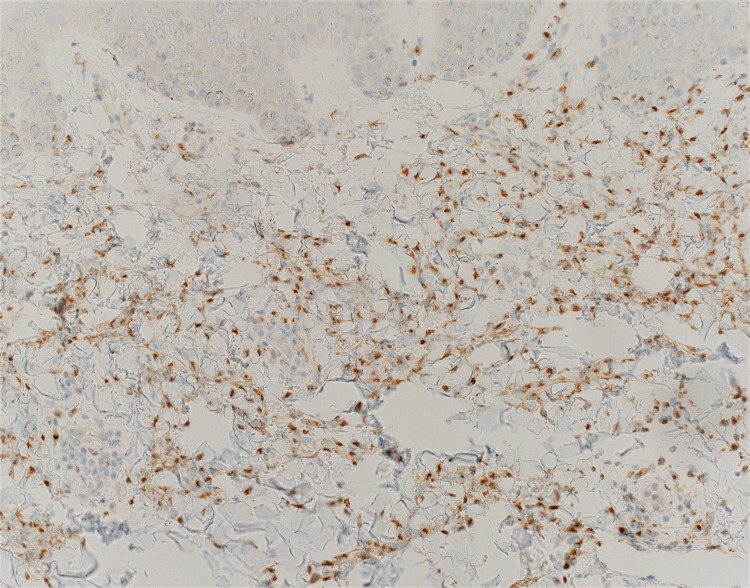
Pathology image identifying the interstitial dermal histiocytic infiltrate staining positively with a CD68 stain, which helps confirm that the involved infiltrates are indeed histiocytes.

Given the dermatological and kidney involvement, our differential diagnosis included urticarial vasculitis, erythema multiforme, and urticaria multiforme. To determine the etiology of the rash, we decided to perform a tangential biopsy of a fresh-looking lesion in the left shoulder after obtaining consent from the patient. We also ordered a urine analysis (UA), urine protein levels, serum levels of C3 and C4, anti-nuclear antibodies, anti-glomerular basement membrane antibodies (anti-GBM), anti-neutrophilic antibodies (ANCA), myeloperoxidase (MPO), viral hepatitis panel, and HIV testing to look for a possible infectious or autoimmune etiology. UA showed significant proteinuria of 2.8 gm in 24 hours, urine microalbumin of 850 mg, and +3 blood but no dysmorphic red blood cells or casts. She had a negative ANA, anti-GBM, viral hepatitis panel, and HIV. She tested positive for P-ANCA (1:640 titer) and MPO, which raised our suspicion for underlying granulomatosis with polyangiitis vasculitis. Her creatinine levels continued to trend upward, reaching a level of 2.5 mg/dl, so an ultrasound-guided renal biopsy was scheduled, and she was started on methylprednisolone 500 mg for three days, followed by a transition to oral prednisone 60 mg once daily. Furthermore, the results of the skin biopsy were complete and showed palisading neutrophilic infiltrates and multiple granulomas, consistent with a diagnosis of interstitial granulomatosis dermatitis (Figures 5-7).

Her skin lesions significantly improved following the methylprednisolone therapy, and her renal biopsy showed focal necrotizing and crescentic pauci-immune rapidly progressive glomerulonephritis. Given the renal biopsy results, the clinical presentation of epistaxis, low-grade fevers, new pulmonary nodules on imaging, and the P-ANCA positivity, a diagnosis of granulomatosis with polyangiitis was given, and the patient was started on rituximab infusions. She completed the first infusion in the inpatient setting and was discharged to complete the rituximab infusions in the outpatient setting and to follow up with the nephrology clinic. 

## Discussion

Granulomatosis with polyangiitis is an uncommon small vessel vasculitis that can present with skin lesions and visceral organ involvement, including mainly the lungs and kidneys [3]. Dermatological manifestations occur in nearly 35% of cases; the most common pathological skin finding is leukocytoclastic vasculitis [4]. Clinically, cutaneous manifestations commonly present as palpable purpura; other skin presentations include dermal nodules, livedo reticularis, pyoderma gangrenosum, and necrotic ulcerations. Cutaneous involvement in granulomatosis with polyangiitis usually presents later during the disease process, but has been reported to present as the initial manifestation of this small vessel vasculitis. Furthermore, skin involvement is more likely to occur with renal involvement [5]. IGD is a rare pathological skin disorder that mainly presents as non-painful and non-pruritic violaceous lesions on the trunk and arms [6]. Histologically, it is characterized by granulomatous inflammation concurring with palisades of histocytes [7]. Although the pathophysiology of this disorder is not clearly understood, it is thought to be most likely secondary to immune complex deposition [8]. IGD is an extremely uncommon pathological skin finding with only 216 cases published in the English literature [9]. In their systematic review, Yang et al. [9] reviewed the characteristics of the 216 reported cases to determine what preceding factors caused IGD in the reported cases. The most common inciting factors were underlying autoimmune disorders (47% of cases), followed by malignancies, drug eruptions, and infectious diseases. Rheumatoid arthritis and systemic lupus erythematosus were the most common autoimmune disorders associated with IGD. Furthermore, there were only six reported cases across the literature of IGD associated with vasculitis as the inciting factor [9]. To the best of our knowledge, our case is the seventh reported case in the literature of IGD associated with a vasculitis disorder and, in particular, granulomatosis with polyangiitis. Our pathologist was able to diagnose IGD by reviewing the underlying literature given its rare nature, and, therefore, it is crucial to report as many cases of IGD as possible, along with their associated inciting disorders, to facilitate easier identification for clinicians in the future. 

## Conclusions

IGD is a very rare dermatological disorder. It has been rarely reported to present with systemic vasculitis such as granulomatosis with polyangiitis. Our pathologist was able to diagnose this disorder after referring to the published literature to confirm the diagnosis. It is vital for clinicians to publish more data and cases regarding IGD to facilitate easier diagnosis among other clinicians given the rarity of this disorder.
